# Climate-mediated evolution of fungicide resistance: insights from interaction among *Phytophthora infestans Cyt-b*_*5*_, azoxystrobin and temperature

**DOI:** 10.1186/s12866-025-04635-8

**Published:** 2025-12-30

**Authors:** Yan-Ping Wang, Li-Na Yang, Fei-Long Xue, Chi Cheng, E-Jiao Wu, Wenjing Wang, Wan-Ying Xie, Xing Li, Songqing Liu, Jiasui Zhan

**Affiliations:** 1 College of Chemistry and Life Sciences, Chengdu Normal University, Chengdu, 611130 China; 2 Sichuan Provincial Key Laboratory for Development and Utilization of Characteristic Horticultural Biological Resources, Chengdu Normal University, Chengdu, 611130 China; 3 Key Laboratory On Conservation and Sustainable Utilization of Marine Biodiversity, Fuzhou Institute of Oceanography, Minjiang University, Fuzhou, 350108 China; 4 Institute of Pomology, Jiangsu Key Laboratory for Horticultural Crop Genetic Improvement,, Jiangsu Academy of Agricultural Sciences, Nanjing, 210014 China; 5 School of Agriculture and Biomanufacturing, Zhengzhou University, Zhengzhou, 450001 China; 6 Department of Forest Mycology and Plant Pathology, Swedish University of Agricultural Sciences, Uppsala, Sweden

**Keywords:** Infectious disease, Pathogen evolution, Fungicide resistance, Climate change, Thermal adaptation, Agricultural sustainability, Genetic diversity, Environment-genotype-chemical interaction

## Abstract

Understanding the spatial variation in the genetic base of fungicide resistance is essential to mitigate the intensifying threats of ongoing climate fluctuation and change to global food security. *Phytophthora infestans*, the causative agent of potato late blight, poses significant challenges to food security due to its rapid adaptation to climate cure and chemical controls. This study explores the role of environmental temperature in shaping the genetic diversity of the cytochrome *b*_*5*_ (*Cyt-b*_*5*_) gene and its association with fungicide tolerance in 96 *P. infestans* from seven different Chinese regions across three climatic zones that vary in their favorability for the development of late blight disease. Analysis of the 96 isolates revealed 13 polymorphic sites in *Cyt-b*_*5*_, yielding nine nucleotide haplotypes and seven amino acid isoforms. Populations from warmer regions exhibited higher genetic diversity, correlating with local temperatures, while cooler regions showed monomorphic haplotypes. Although dependent on specific genotype and stress level, statistical links were observed between *Cyt-b*_*5*_ diversity, both historical and experimental temperatures, and azoxystrobin tolerance, with reduced tolerance and enhanced *Cyt-b*_*5*_ diversity occurring at higher temperatures. These findings underscore temperature as a key driver of genetic variation and suggest its complicated role in resistance dynamics. Warmer climates may enhance mutational diversity, pre-adapting pathogens to evade chemical controls, but also generate genetic load to constrain fitness. The study highlights the need for climate-smart resistance management strategies, integrating regional genetic monitoring and temperature-adjusted fungicide applications to mitigate resistance risks in a warming world.

**Supplementary Information:**

The online version contains supplementary material available at 10.1186/s12866-025-04635-8.

## Introduction

Global food security faces increasing threats from plant diseases, intensified by climate change, shrinking arable land, and rapid population growth. Crop losses due to disease account for 10–15% of pre-harvest food production [1], and even a 50% reduction in these losses could significantly alleviate hunger and malnutrition for billions of people. These challenges are particularly acute in developing countries, where limited resources and technological constraints hinder effective disease management. Fungicides remain a critical tool for controlling plant diseases and safeguarding crop yields. However, the emergence of fungicide resistance poses a major threat to their long-term efficacy. Resistance evolution is shaped by multiple factors, including pathogen biology, fungicide chemistry, application methods and environmental factors [2]. Among environmental drivers, climate plays a pivotal role in disease dynamics, influencing both epidemic outbreaks and long-term pathogen evolution. Temperature is a particularly critical factor, yet the specific impacts of global warming on fungicide resistance remain poorly understood.

As temperatures rise, shifts in pathogen biology, fungicide performance and their interaction are expected, but research on these interactions is scarce. Warmer conditions may accelerate pathogen mutation rates [3], alter fungicide degradation [4], or modify host–pathogen-chemical interactions [5], all of which could influence fungicide efficacy and resistance development. Understanding these mechanisms is essential for designing sustainable disease management strategies in a changing climate. This study investigates the complex interplay among fungicide efficacy, pathogen resistance, and environmental temperature based on *Phytophthora infestans* and quinone outside inhibitor (QoI) interactions. Our research focuses on the response of 96 *P. infestans* isolates collected from China's primary potato production regions representing diverse climatic conditions to QoI stress and the associations of the response to the sequence characters of a Cytochrome b gene and changes of historical and contemporary temperatures.

*P. infestans*, the oomycete pathogen responsible for potato late blight, ranks among the most destructive plant pathogens globally [6]. This organism exhibits remarkable evolutionary capacity, enabling rapid adaptation to both host resistance mechanisms and fungicidal treatments. Characterized by its aggressive spread and destructive potential, *P. infestans* can decimate entire potato crops within days under favorable environmental conditions, particularly in cool, moist climates [7]. The life cycle of the pathogen involves both asexual and sexual reproduction, with airborne sporangia facilitating rapid dispersal across fields [8]. Its ability to overwinter in infected tubers and develop new virulent strains makes it particularly challenging to control, necessitating integrated management strategies combining host resistance and chemical interventions. In developed countries, successful potato cultivation relies on 15–20 fungicide applications per growing season to control late blight [9]. This intensive chemical intervention highlights the critical role of fungicides in modern agriculture, particularly for high-value crops like potatoes. However, the heavy dependence on chemical control raises concerns about long-term sustainability, including the risk of resistance development, environmental impacts, and rising production costs.

QoIs are modern fungicides commonly used to manage plant diseases. They target the cytochrome *bc1* complex at the Qo site within the pathogen’s mitochondrial respiratory chain [10]. This interaction disrupts electron transfer, impairs cellular respiration, and reduces ATP production, ultimately inhibiting fungal growth. Key QoI fungicides have been widely used in agriculture, but their efficacy is increasingly threatened by resistant pathogen strains [11, 12].

Cytochrome *b*_*5*_ (*Cyt-b*_*5*_), a small heme-containing protein in eukaryotes, plays a crucial role in electron transfer and redox reactions [13]. Primarily localized in the endoplasmic reticulum and/or the outer mitochondrial membranes, *Cyt-b*_*5*_ serves as an electron donor for enzymes involved in lipid metabolism, steroid synthesis, and detoxification pathways. Its molecular structure features a characteristic cytochrome fold, with a heme group coordinated by histidine residues around a central iron ion [14]. Through these interactions, *Cyt-b*_*5*_ regulates fatty acid desaturases, supports steroid hormone production, and may contribute to plant secondary metabolite synthesis. Additionally, it collaborates with cytochrome P450 enzymes to enhance the oxidative metabolism of xenobiotics, including fungicides [15].

The development of QoI resistance may involve indirect contributions from *Cyt-b*_*5*_. One proposed mechanism suggests that *Cyt-b*_*5*_ could act as an alternative electron acceptor, bypassing the QoI-inhibited cytochrome *bc1* complex and sustaining energy production in resistant pathogens. Another hypothesis implicates *Cyt-b*_*5*_ in fungicide detoxification, where its partnership with P450 enzymes accelerates QoI degradation, supported by observations that multidrug-resistant pathogens often exhibit expansions in comparison of cytochrome P450 (CYP) genes, with *Cyt-b*_*5*_ as an essential cofactor [16, 17]. Furthermore, *Cyt-b*_*5*_ may contribute to pathogen virulence. For example, *Botrytis cinerea* employs *Cyt-b*_*5*_-dependent systems to counteract host defences such as phytoalexins, while potentially interfering with plant immunity through redox-mediated processes [18]. Despite these insights, the influence of environmental factors, particularly temperature, on QoI resistance evolution remains poorly understood.

As a quinone outside inhibitor, azoxystrobin has served as a cornerstone in late blight management since its introduction in the 1990 s [19]. This broad-spectrum fungicide has demonstrated particular efficacy against *P. infestans*, becoming a key component in anti-blight spray programs worldwide [20, 21]. However, the agricultural community has observed increasing reports of reduced azoxystrobin sensitivity in *P. infestans* populations, with resistance mechanisms primarily involving point mutations in the cytochrome b gene [20]. This emerging resistance threatens the sustainability of current disease management practices, especially considering the limited availability of alternative chemical classes with comparable efficacy and environmental profiles.

Through comprehensive sequencing of the *Cyt-b*_*5*_ gene from different thermal zones and associated phenotypic analyses, we pursue three key specific objectives for the study: first, to elucidate the population genetic structure and evolutionary patterns of *Cyt-b*_*5*_ across geographical populations; second, to assess how local temperature regimes influence the spatial distribution and evolutionary trajectory of resistance-associated alleles; and third, to establish connections between specific genetic variations in *Cyt-b*_*5*_ and fungicide tolerance phenotypes at the population level. These investigations will provide critical insights into how global warming may accelerate fungicide resistance evolution in *P. infestans* and related pathogens*,* informing the development of more sustainable resistance management strategies to protect global agricultural production and food security.

## Materials and methods

### Pathogen collections

*P. infestans* isolates were collected from diseased potato leaves across seven major potato-growing regions in China during the 2010 and 2011 potato-growing seasons: Nanning Guangxi, Fuzhou and Ningde Fujian, Anshun Guizhou, Guyuan Ningxia, Tianshui Gansu, and Kunming Yunnan. These regions collectively accounted for 43.53% of China's total potato harvested area in 2022 (Ministry of Agriculture and Rural Affairs of the People's Republic of China, 2022). Meanwhile, hese regions, separated by 141 to 1,756 km, exhibit significant ecological and agronomic variation, including differences in landscape structure, potato cropping systems, seasonal production frequency, and late blight epidemic patterns [21–24]. The sampled locations represent agroecological zones with varying late blight risks (Table S1). Guizhou and Yunnan, situated in the Southwestern Multiple-cropping Region (SMR), serve as disease epicenters due to high rainfall and climatic conditions that favour annual outbreaks. In contrast, Gansu and Ningxia, located in the drier Northern Single-cropping Region (NSR), experience suboptimal conditions for disease development. Meanwhile, Guangxi, Fuzhou, and Ningde, part of the Southern Winter-cropping Region (SWR), exhibit intermediate late blight prevalence under tropical to subtropical monsoon climates [24].

Infected leaves were randomly sampled from host plants and transported to the laboratory for processing. The infected potato leaves were sampled from commercial fields with permission of growers. Isolates were cultured on rye B agar plates supplemented with ampicillin (100 μg/mL) and rifampicin (10 μg/mL), obtained from sporulating lesion margins. A total of 815 strains were isolated from seven populations. Genotyping was performed using restriction enzyme-PCR amplification of mitochondrial haplotypes, SSR assays of nuclear genomes, and sequence analysis of a housekeeping gene [25–28]. Experimental samples were selected according to the principle of maximizing the diversity of sample genotypes and representing the isolated groups, based on the genotyping. From these analyses, 96 distinct genotypes were selected for further study. Detailed protocols for isolation, purification, and molecular characterization followed established methods [21, 24, 29–31]

### Genomic DNA extraction and *Cyt-b*_*5*_ sequencing

To extract genomic DNA, the 96 *P. infestans* isolates were first retrieved from long-term storage and cultured on rye B agar in the dark at 19 °C for two weeks. The mycelia were harvested, transferred into sterile 2-mL centrifuge tubes, and stored at −80 °C until use. For DNA extraction, the mycelia were ground into a fine powder using liquid nitrogen in a mortar. Genomic DNA from the 96 isolates was then extracted using a plant genomic DNA kit (Promega Biotech. Co., TRANSGEN) following the manufacturer’s instructions. The extracted DNA was stored at −20 °C until further use.

Selective amplification of the *Cyt-b*_*5*_ gene was performed using primers designed from the conserved upstream and downstream regions of the *P. infestans Cyt-b*_*5*_ reference sequence (Genome ID: PITG_08082; Accession No.: NW_003303747.1). The forward primer PiCesaF (CATCCTTCTACACGAGTCGCTACG) and reverse primer PiCesaR (CGCCACCCAATTCTCTTGTCCTT) were used in a 25-µL PCR reaction mixture containing 1.0 µL template DNA, 1.0 µL of each primer (10 µmol/L), 13 µL of 2 × EasyTaq® PCR SuperMix (+ dye), and 9 µL of double-distilled water. The PCR amplification protocol consisted of an initial denaturation step at 94 °C for 5 min, followed by 35 cycles of denaturation at 94 °C for 30 s, annealing at 55 °C for 30 s, and extension at 72 °C for 1 min, with a final extension at 72 °C for 10 min.

The PCR products were separated by electrophoresis on a 1% agarose gel and purified using the QIAquick® Gel Extraction Kit. The purified DNA was ligated into the T5 Zero Cloning Vector and transformed into Trans1-T1 competent cells via heat shock at 42 °C for 30 s using the pEASY®-T5 Zero Cloning Kit. Colonies containing inserts of the expected size were selected and sent to Beijing Tsingke Biotech Co., Ltd. for sequencing using an ABI3730 DNA Analyzer.

### Azoxystrobin tolerance assay

Azoxystrobin tolerance data were retrieved from a previous publication [30], with the detailed experimental protocol fully described therein. Briefly, experimental isolates were recovered from long-term storage at 4 °C and cultured on rye B agar medium at 19 °C for 8 days prior to testing. A stock solution of azoxystrobin was prepared by dissolving the fungicide in methanol, which was subsequently diluted to the required concentrations for experimentation. Azoxystrobin tolerance was calculated as the relative growth rate in fungicide-treated samples compared to untreated controls at five temperatures (13, 16, 19, 22 and 25 °C) with three replications, following established protocols [24, 32]. The experimental concentrations of azoxystrobin were 0.05, 0.10 and 0.30 μg/ml, with parallel control treatments (without azoxystrobin) included for comparison. Colony sizes of both treated and control isolates were digitally recorded daily for one week, with subsequent image analysis performed using specialized software to estimate growth rates using a logistic model [33].

### Data analysis

Nucleotide sequence peaks were visually inspected using Chromas (https://technelysium.com.au/wp/chromas/) and manually verified for accuracy [34, 35]. Nucleotide composition analysis was performed using the BioEdit Sequence Alignment Editor [36]. Amino acid sequences were deduced through codon-based translation of nucleotide triplets using the EXPASy translation tool. Multiple sequence alignment of the *Cyt-b*_*5*_ gene was conducted using ClustalW implemented in MEGA 11.0.10 [37], with mutation site mapping performed using BioEdit. Haplotype construction for both nucleotide and amino acid sequences was carried out using the PHASE algorithm in DNA Sequence Polymorphism version 6.11.01 [38].

Genetic variation within *P. infestans* populations was assessed through multiple parameters including nucleotide diversity, haplotype diversity, the average number of nucleotide differences and haplotype richness. These analyses were performed for individual populations as well as combined populations, with sequences pooled according to host origin and geographical location using DnaSP 6. A median-joining haplotype network was generated in DnaSP6 and visualized using PopART 1.7 [39], where each haplotype was represented by a circle with size proportional to its frequency among isolates. Nucleotide substitutions between haplotypes were indicated by tick marks. Phylogenetic reconstruction was performed on both unique *Cyt-b*_*5*_ haplotypes and complete sequence sets using the neighbour-joining method in MEGA 7.0.21 [40], with tree robustness assessed through 1,000 bootstrap replicates.

Statistical analyses included χ^2^ tests to evaluate homogeneity in *Cyt-b*_*5*_ sequence nucleotide proportions, haplotype frequencies, and isoform frequencies among populations [41]. Amino acid isoforms were determined using MEGA 7.0.21 and visualized with ESPript (http://espript.ibcp.fr/ESPript/ESPript/). These isoforms were designated with "AAH" prefixes followed by numerical identifiers, with subsequent amino acid composition analysis performed in BioEdit. The Cyt-b_5_ protein structure homology-modelling was built using SWISS-MODEL Server [42]. Analysis of variance (ANOVA) for azoxystrobin tolerance among Cyt-b_5_ isoforms and *P. infestans isolates* population was conducted using SPSS software [43]. Differences in tolerance levels were further examined using Duncan's Multiple Range Test for post-hoc comparisons [44]. Pearson correlation analysis [45] was employed to examine key relationships, particularly the association of genetic variation in the *Cyt-b*_*5*_ gene with azoxystrobin mean tolerance of *P. infestans* and long-term (20-year average) annual mean temperatures at collection sites, with climate data obtained as previously described [24, 46].

## Results

### Primary structure of *Cyt-b*_*5*_

Full *Cyt-b*_*5*_ sequences were obtained from all 96 *P. infestans* isolates across the seven populations. The sequences were identical in length, as no deletions, insertions, duplications, or premature termination events were detected in the *Cyt-b*_*5*_ gene. The gene, an intron-free sequence of 444 nucleotides, encoded a protein of 147 amino acids with a mean molecular weight of 135,414.92 Daltons. Nucleotide composition analysis showed an average distribution of 17.34% adenine, 22.51% thymine, 31.35% cytosine, and 28.80% guanine (Fig. S1). Statistical analyses confirmed that the base composition significantly deviated from the theoretical expectation of equal nucleotide proportions, with a GC content (60.15%) markedly higher than the AT content (χ^2^ = 82.48, df = 1, *P* < 0.0001), indicating a strong compositional bias in the gene.

### Mechanisms of genetic variation generation

Multiple sequence alignment demonstrated that all sequence variations in *Cyt-b*_*5*_ resulted from point mutations, with 13 polymorphic sites identified, producing nine distinct nucleotide haplotypes (Table 1). Among these, eight mutations were transversions, and five were transitions (Table 2). The dominant nucleotide haplotype, H1, matched the reference strain’s gene structure and accounted for 86.46% of the population (Table 3). Compared to H1, nucleotide haplotypes H2–H4 and H8 exhibited a transition mutation (A → G) and a transversion (C → G), while H4 and H7 carried a transversion (T → G). Additionally, H2 and H4 displayed transversion mutations (A ↔ C), while transition mutations (C ↔ T) were observed in haplotypes H2–H6 and H8–H9.

**Table 1:** Sample sizes, number of segregating sites, haplotypes, and diversity indices (haplotype diversity, average number of nucleotide differences, and nucleotide diversity) for *Cyt-b*_*5*_ gene in *P. infestans* populations from different geographic regions

Population	Haplotypes	Number of sequences	Number of segregating sites	Number of haplotypes	Haplotype diversity	Average number of differences	Nucleotide diversity
Guizhou	H1	18	0	1	0	0	0
Fuzhou	H1-3	13	5	3	0.4103	1.6923	0.0038
Guangxi	H1-4	8	11	4	0.6429	3.8929	0.0088
Gansu	H1, H5-7	16	4	4	0.35	0.6083	0.0014
Ningxia	H1	15	0	1	0	0	0
Ningde	H1, H6 H8-9	15	5	4	0.4667	0.9905	0.0022
Yunnan	H1	11	0	1	0	0	0
Total	H1-H9	96	13	9	0.2522	0.8783	0.002

H1-H9 represent distinct nucleotide haplotypes

**Table 2:** Variation sites and types of *Cyt-b*_*5*_ gene in the 96 sequences

Positions and types of substitution	T30-4	H1	H2	H3	H4	H5	H6	H7	H8	H9
41v	C	C	C	C	G	C	C	C	G	C
71v	T	T	T	T	G	T	T	T	T	T
88v	A	A	A	A	C	A	A	A	A	A
107v	T	T	T	T	G	T	T	T	T	T
141 s	C	C	C	C	C	T	C	C	C	C
197v	C	C	C	C	G	C	C	C	C	C
223 s	G	G	G	G	A	G	G	G	G	G
270 s	C	C	T	T	T	T	T	C	T	C
310 s	A	A	G	G	G	A	A	A	G	A
321v	C	C	G	G	G	C	C	C	G	C
382v	C	C	A	C	C	C	C	C	C	C
410v	T	T	T	T	T	T	T	G	T	T
413 s	T	T	C	C	C	C	T	T	C	C

Nucleotide variations at 13 polymorphic sites across nine *Cyt-b*_*5*_ haplotypes (H1-H9) compared to reference strain T30-4. 's' denotes transition mutations; 'v' denotes transversion mutations

**Table 3:** Frequency of nucleotide haplotypes (H1-H9) and corresponding amino acid haplotypes (AAH1-AAH7) in each geographic population

Amino acid haplotype	Nucleotide haplotypes	Population						
		Fuzhou	Ningde	Guangxi	Yunnan	Gansu	Ningxia	Guizhou
AAH1	H1,H6	10	13	5	11	14	15	18
AAH2	H2	1	0	1	0	0	0	0
AAH3	H3	2	0	1	0	0	0	0
AAH4	H4	0	0	1	0	0	0	0
AAH5	H5,H9	0	1	0	0	1	0	0
AAH6	H7	0	0	0	0	1	0	0
AAH7	H8	0	1	0	0	0	0	0

AAH1 represents the reference protein sequence

### Haplotype network and phylogenetic tree of *Cyt-b*_*5*_ gene

**Fig. 1:**
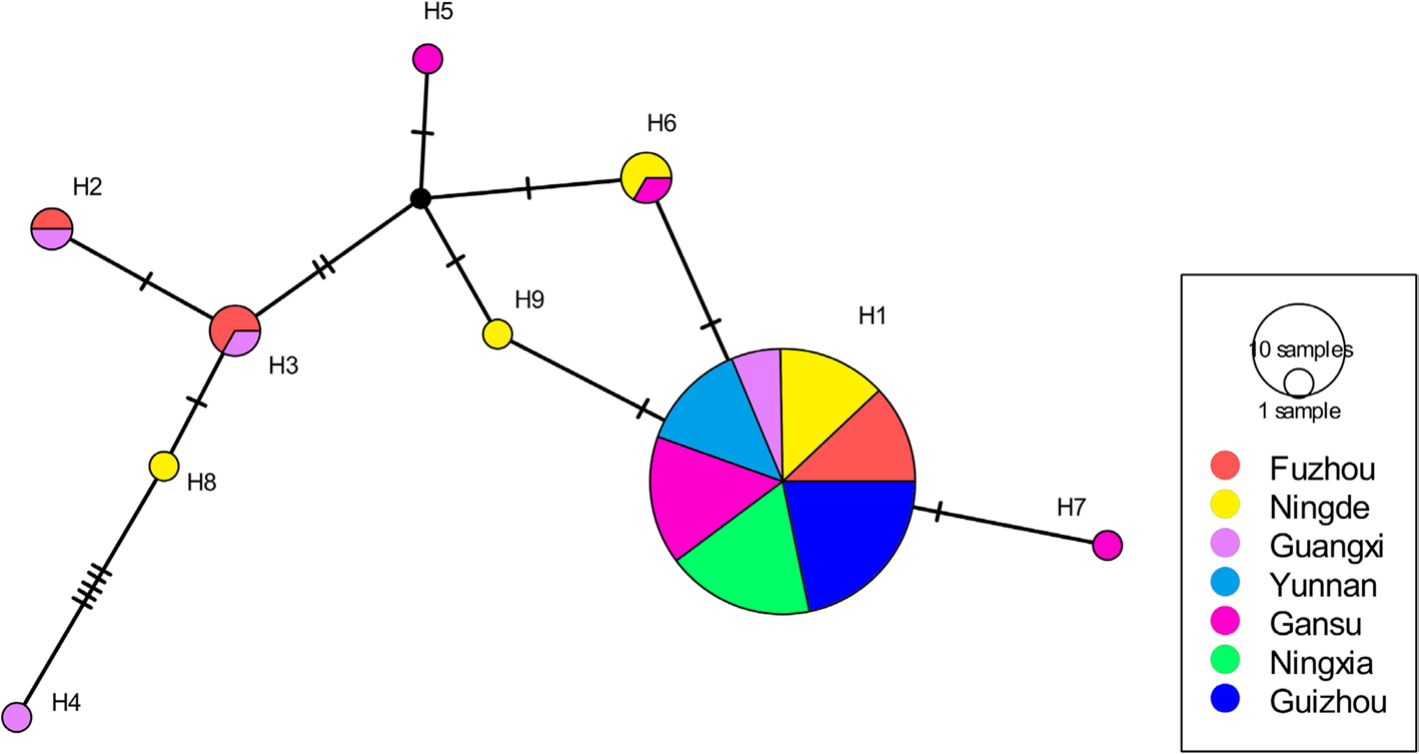
Median-joining network of *Cyt-b*_*5*_ nucleotide haplotypes from seven Chinese *P. infestans* populations. Circle sizes correspond to haplotype frequencies in the pooled sample, with tick marks indicating mutational steps between haplotypes. Black dots represent hypothetical intermediate haplotypes not detected in our sampling. The network reveals two distinct haplotype groups separated by a missing intermediate, with maximum divergence of 11 mutation steps between H7 and H4 haplotypes

The nucleotide haplotypes of *Cyt-b*_*5*_ exhibited a maximum divergence of 11 mutation steps, observed between H7 and H4 (Fig. 1). The haplotype network revealed a missing intermediate nucleotide haplotype that divided the nucleotide haplotypes into two distinct groups. The first group consisted of four nucleotide haplotypes (H1, H6, H7, and H9), differing by 1–2 mutation steps and forming a reticulate structure with the missing nucleotide haplotype. The second group included five nucleotide haplotypes (H2–H5 and H8), separated by 1–9 mutational steps. Among these, H4 diverged from H8 by five steps, while the remaining haplotypes were 1–3 mutation steps apart from their nearest neighbours.

**Fig. 2:**
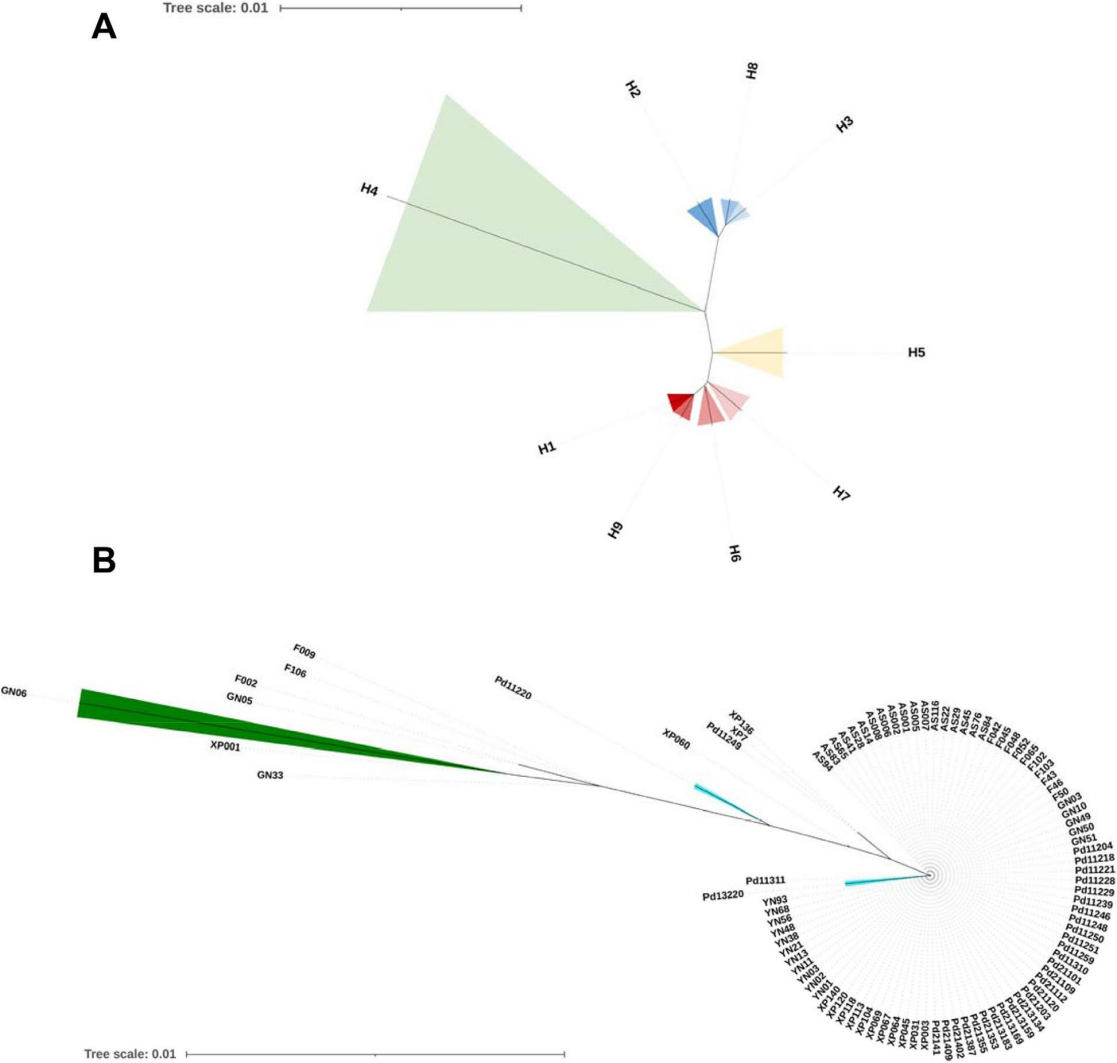
Phylogenetic reconstruction of *Cyt-b*_*5*_ sequences using Maximum Composite Likelihood method in MEGA 11. Panel A shows the relationships among nine nucleotide haplotypes, while Panel B displays the complete tree including all 96 sequences from seven sub-populations. Bootstrap values exceeding 70% are indicated at branch nodes, with scale bars representing nucleotide substitutions per site. The topology confirms the genetic divergence observed in the haplotype network, with four major branches corresponding to distinct haplotype clusters

Consistent with the network results, Neighbour-Joining (NJ) phylogenetic analysis grouped the 96 *Cyt-b*_*5*_ sequences into four major branches (Fig. 2). The rare H4 and H8 haplotype, represented by a single Guangxi isolate (GN06) and a single Ningde isolate (XP001) separately, with H8 further forming a separate sub-clade, formed an independent branch and were the most genetically distant from the dominant H1 haplotype. Another branch comprised two H2 sequences and three H3 sequences, with H2 clustering into a sub-clade. Similarly, the H5 haplotype from a Gansu isolate (Pd11220) and the H9 haplotype from a Ningde isolate (XP60) also appeared as a distinct branch separately. The H6 haplotype nearest to the dominant haplotype H1 was composed of three samples from a Guangxi isolate (Pd11249) and two Ningde isolates (XP7 and XP136). The largest branch included 83 H1 sequences and one H7 sequence (Pd13220), with H7 further forming a separate sub-clade.

### Spatial distribution and population genetic diversity in *Cyt-b*_*5*_

The *Cyt-b*_*5*_ gene displayed distinct patterns of genetic diversity across the seven *P. infestans* populations. The average number of nucleotide differences ranged from 0.0000 to 3.8929, with an overall difference of 0.8783 in the pooled dataset (Table 1). Nucleotide diversity varied from 0.0000 to 0.0088, yielding a total diversity of 0.0020, while haplotype diversity spanned from 0.0000 to 0.6429, culminating in a combined population diversity of 0.2522. The Guangxi population exhibited the highest genetic diversity, containing 11 segregating sites, four haplotypes (H1–H4), a haplotype diversity of 0.6429, and the highest average nucleotide differences (3.8929) and nucleotide diversity (0.0088). In contrast, populations from Guizhou, Ningxia, and Yunnan showed no genetic variation, with only the dominant haplotype (H1) present, resulting in zero haplotype and nucleotide diversity. Intermediate diversity levels were observed in the Fuzhou and Ningde populations, which contained 3–5 segregating sites and haplotype diversities of 0.4103 and 0.4667, respectively. Although the Gansu population harboured four haplotypes (H1, H5–H7), its diversity metrics (haplotype diversity = 0.35, nucleotide diversity = 0.0014) were lower than those of Fuzhou and Ningde. These results underscore substantial regional differences in *Cyt-b_*5*_ * variation, with Guangxi emerging as a diversity hotspot, while Guizhou, Ningxia, and Yunnan populations remained genetically uniform.

### Genetic variation in the deduced isoform of *P. infestans Cyt-b*_*5*_

**Fig. 3:**
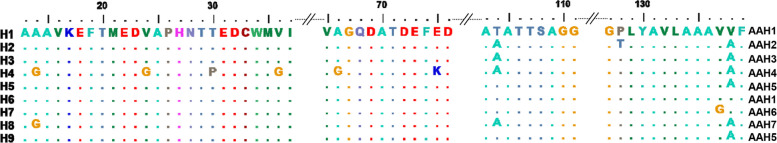
Multiple sequence alignment of variable Cyt-b_5_ amino acid residues deduced from nucleotide haplotypes. The consensus sequence (Global score > 0.70) appears in the first line, with dots indicating identity to the reference AAH1 haplotype. Mutated residues are annotated with position numbers and amino acid changes. The alignment was generated using ClustalW and visualized in BioEdit, revealing seven distinct amino acid haplotypes resulting from non-synonymous mutations

Two *Cyt-b*_*5*_ nucleotide haplotypes, including the dominant H1 and one additional nucleotide haplotype (H6), translated into amino acid haplotype 1 (AAH1) (Table 3, Fig. 3). AAH1 was present in all *P. infestans* sub-populations and was the sole isoform detected in Ningxia, Guizhou, and Yunnan (100%, Table 3). Meanwhile, amino acid haplotype 5 (AAH5) was deduced by two nucleotide haplotypes (H5 and H9). The remaining five nucleotide haplotypes (H2–H4, H7–H8) encoded five distinct amino acid haplotypes (AAH2–AAH4, AAH6-AAH7).

H5 and H9 contained a T → C mutation at nucleotide position 413, converting valine to alanine at residue 138 A138V (AAH5) which were restricted to Ningde and Gansu populations. H7 featured a C → G mutation at position 410, resulting in a valine-to-glycine substitution at residue 137 G137V (AAH6). Compared to H5, H3 carried an A → G mutation at position 310, changing threonine to alanine at residue 104 A104T (AAH3). Relative to H3 and H5, H2 had an additional C → A mutation at position 382, altering proline to threonine at residue 128 T128P (AAH2). Similarly, H8 possessed a C → G mutation at position 41, substituting alanine with glycine at residue 14 G14A (AAH7). H4 exhibited five additional mutations (T → G, A → C, T → G, C → G, G → A at positions 71, 88, 107, 197 and 223) in the *Cyt-b*_*5*_ domain at position 19 to 96 (Fig. S2), leading to valine → glycine, threonine → proline, valine → glycine, alanine → glycine and Glutamic acid → Lysine changes at residues 24, 30, 36, 66 and 75, respectively (AAH4, G24V, P30T, G36V, G66A, K75E). AAH2 and AAH3 were restricted to Fuzhou and Guangxi populations, while the rare AAH4, AAH6-7 variants were exclusively found in Guangxi, Gansu (two haplotypes), and Ningde populations (Table 3).

### Association among azoxystrobin tolerance, genetic variation of *Cyt-b*_*5*_ genes, and local air temperature

**Fig. 4:**
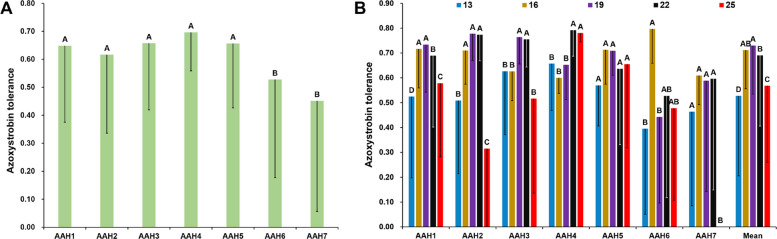
Azoxystrobin tolerance of 96 *Phytophthora infestans* isolates sampling from seven geographic locations carrying different Cyt-b_5_ amino acid haplotypes (AAH1-AAH7). **A** the average azoxystrobin tolerance of AAHs estimated with five temperatures (13, 16, 19, 22 and 25 °C) at three experimental concentrations of azoxystrobin (0.05, 0.10 and 0.30 μg/ml). **B** the azoxystrobin tolerance of AAHs under five temperatures (13, 16, 19, 22 and 25 °C) estimated with three experimental concentrations of azoxystrobin (0.05, 0.10 and 0.30 μg/ml). Its error bars were computed across all isolates in each population under the three azoxystrobin concentrations. (0.05, 0.10, and0.30 μg/ml). Duncan’s multiple range test for differences in azoxystrobin tolerance among different Cyt-b_5_ amino acid haplotypes. Values followed by different letters in the same column are significantly different at *P* = 0.05

**Fig. 5:**
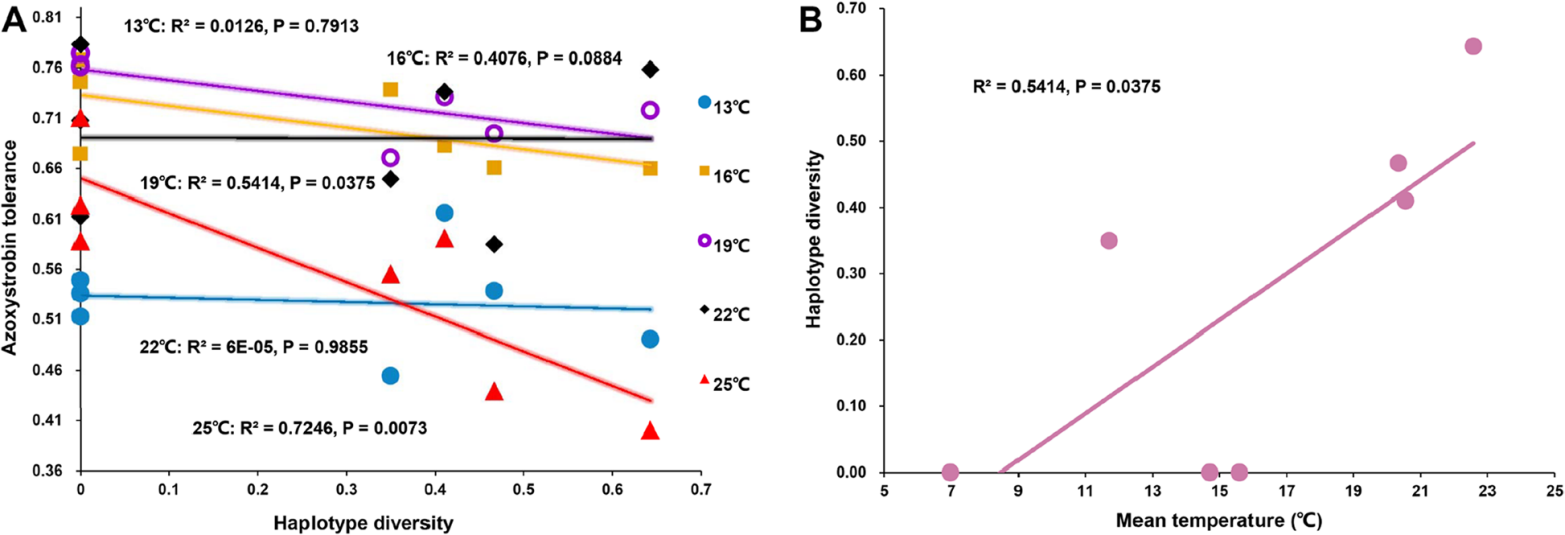
Bivariate correlations examining relationships between **A**) azoxystrobin tolerance and *Cyt-b*_*5*_ haplotype diversity, **B**) mean annual temperature and *Cyt-b*_*5*_ haplotype diversity. Regression lines with Pearson correlation coefficients (r) demonstrate a negative relationship between fungicide tolerance and genetic diversity, while temperature shows positive associations with *Cyt-b*_*5*_ variation

The relationship between *Cyt-b*_*5*_ genetic variation, local air temperature, and fungicide response was investigated through temperature-dependent assays. Statistically significant differences in azoxystrobin tolerance were observed among Cyt-b_5_ isoforms, and the experimental temperatures (Fig. 4 and Fig. S3). Notably, AAH2 and AAH3 exhibited a 10–20% reduction in tolerance at 25 °C compared to 19 °C. In contrast to other AAHs, which generally performed best at 19 °C, AAH4 showed marginally higher azoxystrobin tolerance at 22 °C and 25 °C, and AAH6 showed marginally higher azoxystrobin tolerance at 16 °C (Fig. 4B). Further analysis revealed that genetic diversity, both in nucleotide haplotypes and amino acid isoforms, was negatively correlated with azoxystrobin tolerance particularly under heat stress (25 °C) but positively associated with local air temperature (Fig. 5 and Fig. S4).

## Discussion

This study investigated the complex interplay between genetic variation in the *Cyt-b*_*5*_ gene, fungicide tolerance, and environmental temperature in *P. infestans* populations originating from diverse agroecological regions across China. Our results demonstrates that environmental temperature is a key driver of genetic diversity in the *Cyt-b5* gene of *P. infestans*, which in turn influences the pathogen's tolerance to the fungicide azoxystrobin in a temperature-dependent manner. Our key findings are threefold: First, populations from warmer regions exhibited significantly higher *Cyt-b5* genetic diversity than those from cooler areas. Second, this increased genetic diversity was negatively correlated with azoxystrobin tolerance under heat stress, contradicting the simple expectation that higher diversity invariably facilitates adaptation. Third, the relationship between genotype and phenotype is complex and modulated by experimental temperature. These findings suggest that temperature plays a dual role in the evolutionary development of fungicide resistance in *P. infestans* and mutations in the *Cyt-b*_*5*_ domain region may contribute to the increased azoxystrobin tolerance of *P. infestans* at higher temperatures as documented previously in the association analysis between cytochrome b mutations in QoI resistance [47]. At the genetic level, temperature appears to reshape the population structure of this key fungicide target gene through selection pressures that may influence mutation rates or maintenance of genetic variation. At the epigenetic level, temperature fluctuations could modulate gene expression patterns, potentially altering the pathogen's response to fungicide exposure. This temperature-mediated influence on both genetic and epigenetic regulation of *Cyt-b*_*5*_ aligns with emerging evidence about the eco-evolutionary role of climate factors, particularly temperature, in shaping functional traits across species [48].

The spatial distribution of genetic variation in *Cyt-b*_*5*_ was strongly correlated with local thermal regimes, revealing a counterintuitive pattern. Populations from warmer regions (e.g., Guangxi, Fuzhou and Ningde) displayed higher genetic diversity than those from cooler areas (e.g., Ningxia, Gansu and Yunnan), as supported by a significantly positive correlation between mean annual temperature and haplotype diversity. This finding appears counterintuitive. With smaller potato cultivation areas (< 100 k hectares) and less conducive conditions for late blight epidemics, pathogen populations in Guangxi, Fuzhou, and Ningde were expected to harbor lower genetic diversity compared to those in larger production regions (> 500 k hectares) like Yunnan, Gansu and Ningxia, where epidemic pressure is also higher [49]. We propose two non-mutually exclusive mechanisms to explain this discrepancy. First, human-mediated gene flow is a highly likely factor. In SWR, the common practice of the common practice of importing seed potatoes imported from NSR and other regions [50], consistently introduces diverse pathogen strains, artificially elevating local haplotype diversity, repair efficiency, consistent with the "more mutations" hypothesis [51]. Second, temperature-dependent mutagenesis offers a plausible evolutionary explanation, whereby warmer climates may increase replication errors and reduce DNA repair efficiency, as per the "more mutations" hypothesis [52]. The high *Cyt-b5* diversity in these warmer regions likely stems from a combination of both mechanisms: human activity facilitates the introduction of genetic variation, while elevated temperatures may accelerate its generation and maintenance.

The observed genetic variation in *Cyt-b*_*5*_ was primarily driven by point mutations, with limited evidence of recombination suggested by the reticular structure in the haplotype network. This is consistent to the previous finding on the genetic variation of the gene [31] but contrasts sharply with effector genes from the same pathogen isolates, which often evolve through diverse mechanisms including insertions, deletions, premature stop codons, or absent start codons to generate rapid variation and evade host immunity [53]. The conservative evolutionary trajectory of *Cyt-b*_*5*_ aligns with its essential role for electron transfer and lipid metabolism. This underscores the distinct evolutionary constraints acting on pathogen genes: while effector genes require genetic flexibility to overcome host defenses, *Cyt-b*_*5*_ is likely under stabilizing selection to maintain critical functions, resulting in slower, mutation-driven evolution. Such differences highlight how gene function dictates evolutionary tempo and mode, with implications for understanding adaptation in both pathogenic and non-pathogenic contexts.

Fisher’s fundamental theorem posits that adaptation is most efficient in high-variance populations [54]. However, the observed negative correlation between diversity and tolerance contradicts this hypothesis. In the case of *Cyt-b*_*5*_, stabilizing selection driven by functional trade-offs or pleiotropy [55] may explain this discrepancy. In this case, high genetic variation can impose a genetic load [56] and some mutations in genetically diverse populations from warm regions may be deleterious, reducing overall fitness of species and diminishing fungicide adaptation efficiency. Alternatively, diversifying selection in warm climates could maintain alleles that are suboptimal for fungicide resistance but advantageous in other ecological contexts, reflecting trade-offs [57]. Thus, populations with greater genetic diversity may face fitness compromises, limiting their adaptability to chemical stressors. This inconsistency might also arise from functional redundancy in the mitochondrial respiratory chain, where alternative electron transfer pathways compensate for *Cyt-b*_*5*_ mutations. Additionally, *Cyt-b*_*5*_’s role could be context-dependent [13, 58], requiring specific genetic or environmental conditions for phenotypic effects to emerge. Empirical support for such constraints is well-documented in other microbial pathogens. For instance, in the wheat pathogen *Zymoseptoria tritici*, fungicide resistance mutations often carry a significant fitness cost in the absence of the chemical, a trade-off that helps maintain susceptibility alleles in populations [59]. Similarly, in *Mycobacterium tuberculosis*, the fitness burden (genetic load) imposed by drug-resistance mutations frequently necessitates compensatory evolution to restore viability [60].

Collectively, these results highlight the pivotal role of local temperature in shaping the genetic structure of *P. infestans* populations, with warmer regions exhibiting higher genetic diversity due to ecological and evolutionary mechanisms. The observed temperature-genotype–phenotype relationships provide critical insights into how fungicide resistance may evolve under climate change. As global temperatures rise, shifts in thermal regimes could alter the genetic architecture of fungicide target genes in pathogen populations, potentially accelerating resistance in some regions while creating unexpected susceptibility patterns in others, consistent with broader ecological-evolutionary principles, where thermal gradients drive local adaptation in microbial populations [61]. These findings also reinforce the growing evidence that climate variability complicates disease management strategies [49].

Based on our findings, several specific, climate-smart strategies can be proposed for managing fungicide resistance. First, regional genetic monitoring of key fungicide target genes like *Cyt-b*_*5*_ should be implemented. Identifying regions with high genetic diversity can help predict potential resistance hotspots before they lead to control failures. Second, temperature-adjusted fungicide application protocols could be developed. For example, in warmer regions or during hotter seasons, the timing and frequency of QoI applications might need adjustment, potentially integrating them with other management tools to reduce selection pressure. Third, the strong negative correlation between diversity and tolerance in warm areas underscores the importance of diversifying selection pressures. This reinforces the critical need for mixtures or rotations of fungicides with different modes of action to ensure that pathogen populations with high genetic diversity are not consistently selected for resistance to a single chemical class. Finally, deploying resistant potato cultivars in tandem with chemical controls will be essential to provide a dual barrier against this rapidly evolving pathogen. However, this study has limitations. Our findings, while revealing strong correlations, stop short of establishing causality, as the functional consequences of the identified *Cyt-b5* mutations remain unverified. Furthermore, broader sampling across a wider range of climates and host plants would strengthen the generalizability of our conclusions. Future research should therefore integrate functional genomics with landscape epidemiology to fully unravel the genetics-environment-management interplay. A critical next step is the experimental validation of these mutations. Employing modern genetic tools such as CRISPR-Cas9-mediated gene editing to introduce specific point mutations into a common genetic background or heterologous expression of variant *Cyt-b*_*5*_ alleles in a model system would establish a direct causal link. Such functional analyses are essential to determine unequivocally whether these mutations are adaptive, neutral, or deleterious under different temperature regimes, thereby elucidating the mechanistic basis of *Cyt-b5's* role in QoI response.

## Supplementary Information

Supplementary Material 1. Table S1. Geographic and environmental information of seven potato growing areas.

Supplementary Material 2. Fig. S1. Nucleotide composition analysis of the 444-bp Cyt-b_5_coding region across 96 P. infestans isolates. The histogram displays percentage distributions of adenine (A, 17.34%), thymine (T, 22.51%), cytosine (C, 31.35%), and guanine (G, 28.80%), with error bars representing standard deviations. The dashed line indicates theoretical equal distribution (25% per base). Statistical analysis confirms significant GC bias (60.15% GC content; χ² test, *P* < 0.0001) deviating from random expectation. Fig. S2 Visualization of modeled tertiary structure of Cyt-b_5_ generated by SWISS-MODEL Workspace. The Cyt-b_5_ domain are located in 19 to 96 amino acid residues labeled as a cyan background. 46 PDB ID: 4V7E was having a maximum sequence identity of 19.67%. The used template (AlphaFold DB model of A0A3R7JV96_9STRA) was having a maximum sequence identity of 78.08%. Fig. S3. Azoxystrobin tolerance of 96 *Phytophthora infestans* isolates sampling from seven geographic locations carrying different Cyt-b_5_amino acid haplotypes (AAH1-AAH7). A) 0.05 azoxystrobin, B) 0.10 μg/ml azoxystrobin, C) 0.30 μg/ml azoxystrobin. Its error bars were computed across all isolates in each population under the three azoxystrobin concentrations. (0.05, 0.10, and0.30 μg/ml). Duncan’s multiple range test for differences in azoxystrobin tolerance among different Cyt-b_5_amino acid haplotypes. Values followed by different letters in the same column are significantly different at *P*= 0.05. Fig. S4. Bivariate correlations examining relationships between mean annual temperature and Cyt-b_5_isoform diversity across seven populations. A) 0.05μg/ml azoxystrobin tolerance and Cyt-b_5_haplotype diversity, B) 0.10μg/ml azoxystrobin tolerance and Cyt-b_5_haplotype diversity, C) 0.30μg/ml azoxystrobin tolerance and Cyt-b_5_haplotype diversity, D) mean annual temperature and Cyt-b_5_ amino acid haplotype diversity. Regression lines with Pearson correlation coefficients (r) demonstrate a negative relationship between fungicide tolerance and genetic diversity, while temperature shows positive associations with Cyt-b_5_variation.

## Data Availability

Associated Cyt-b5 gene sequences data during the current study have been deposited in GenBank: Accession Numbers PX244718-PX244813.
