# Supplementary material for: Climate-mediated evolution of fungicide resistance: insights from interaction among Phytophthora infestans Cyt-b5, azoxystrobin and temperature
Source: BMC Microbiol. 2025 Dec 30;26:75. doi: 10.1186/s12866-025-04635-8 (PMC12860030; doi:10.1186/s12866-025-04635-8)
Supplement: Supplementary file 1 — Supplementary Material 1. Table S1. Geographic and environmental information of seven potato growing areas. [file 12866_2025_4635_MOESM1_ESM.docx]

**Table S1** Geographic and environmental information of seven potato growing areas

| Population | Longitude | Latitude | Altitude (m) | Annual temperature  (°C) | Annual rainfall (mm) | Potato cultivation pattern | Climate conditions |
| --- | --- | --- | --- | --- | --- | --- | --- |
| Fuzhou Fujian | 119°17' | 26°05' | 10 | 20.54 | 1500-1713 | Southern Winter-cropping Region (SWR) | subtropical |
| Tianshui Gansu | 105°43' | 34°35' | 2089 | 11.71 | 470-610 | Northern Single-cropping Region (NSR) | continental |
| Nanning Guangxi | 108°22' | 22°50' | 78 | 22.58 | 1250-1750 | Southern Winter-cropping Region (SWR) | subtropical |
| Anshun Guizhou | 105°56' | 26°16' | 1330 | 14.71 | 1000-1400 | Southwestern Multiple-cropping Region (SMR) | temperate |
| Guyuan Ningxia | 106°14' | 36°01' | 1778 | 6.96 | 167-618 | Northern Single-cropping Region (NSR) | continental |
| Ningde Fujian | 119°59' | 26°54' | 31 | 20.33 | 1600-2200 | Southern Winter-cropping Region (SWR) | subtropical |
| Kunming Yunnan | 102°43' | 25°03' | 2677 | 15.6 | 550-760 | Southwestern Multiple-cropping Region (SMR) | temperate |
