# Supplementary material for: Climate-mediated evolution of fungicide resistance: insights from interaction among Phytophthora infestans Cyt-b5, azoxystrobin and temperature
Source: BMC Microbiol. 2025 Dec 30;26:75. doi: 10.1186/s12866-025-04635-8 (PMC12860030; doi:10.1186/s12866-025-04635-8)
Supplement: Supplementary file 2 — Supplementary Material 2. Fig. S1. Nucleotide composition analysis of the 444-bp Cyt-b5coding region across 96 P. infestans isolates. The histogram displays percentage distributions of adenine (A, 17.34%), thymine (T, 22.51%), cytosine (C, 31.35%), and guanine (G, 28.80%), with error bars representing standard deviations. The dashed line indicates theoretical equal distribution (25% per base). Statistical analysis confirms significant GC bias (60.15% GC content; χ² test, P < 0.0001) deviating from random expectation. Fig. S2 Visualization of modeled tertiary structure of Cyt-b5 generated by SWISS-MODEL Workspace. The Cyt-b5 domain are located in 19 to 96 amino acid residues labeled as a cyan background. 46 PDB ID: 4V7E was having a maximum sequence identity of 19.67%. The used template (AlphaFold DB model of A0A3R7JV96_9STRA) was having a maximum sequence identity of 78.08%. Fig. S3. Azoxystrobin tolerance of 96 Phytophthora infestans isolates sampling from seven geographic locations carrying different Cyt-b5amino acid haplotypes (AAH1-AAH7). A) 0.05 azoxystrobin, B) 0.10 μg/ml azoxystrobin, C) 0.30 μg/ml azoxystrobin. Its error bars were computed across all isolates in each population under the three azoxystrobin concentrations. (0.05, 0.10, and0.30 μg/ml). Duncan’s multiple range test for differences in azoxystrobin tolerance among different Cyt-b5amino acid haplotypes. Values followed by different letters in the same column are significantly different at P= 0.05. Fig. S4. Bivariate correlations examining relationships between mean annual temperature and Cyt-b5isoform diversity across seven populations. A) 0.05μg/ml azoxystrobin tolerance and Cyt-b5haplotype diversity, B) 0.10μg/ml azoxystrobin tolerance and Cyt-b5haplotype diversity, C) 0.30μg/ml azoxystrobin tolerance and Cyt-b5haplotype diversity, D) mean annual temperature and Cyt-b5 amino acid haplotype diversity. Regression lines with Pearson correlation coefficients (r) demonstrate a negative relationship b [file 12866_2025_4635_MOESM2_ESM.docx]

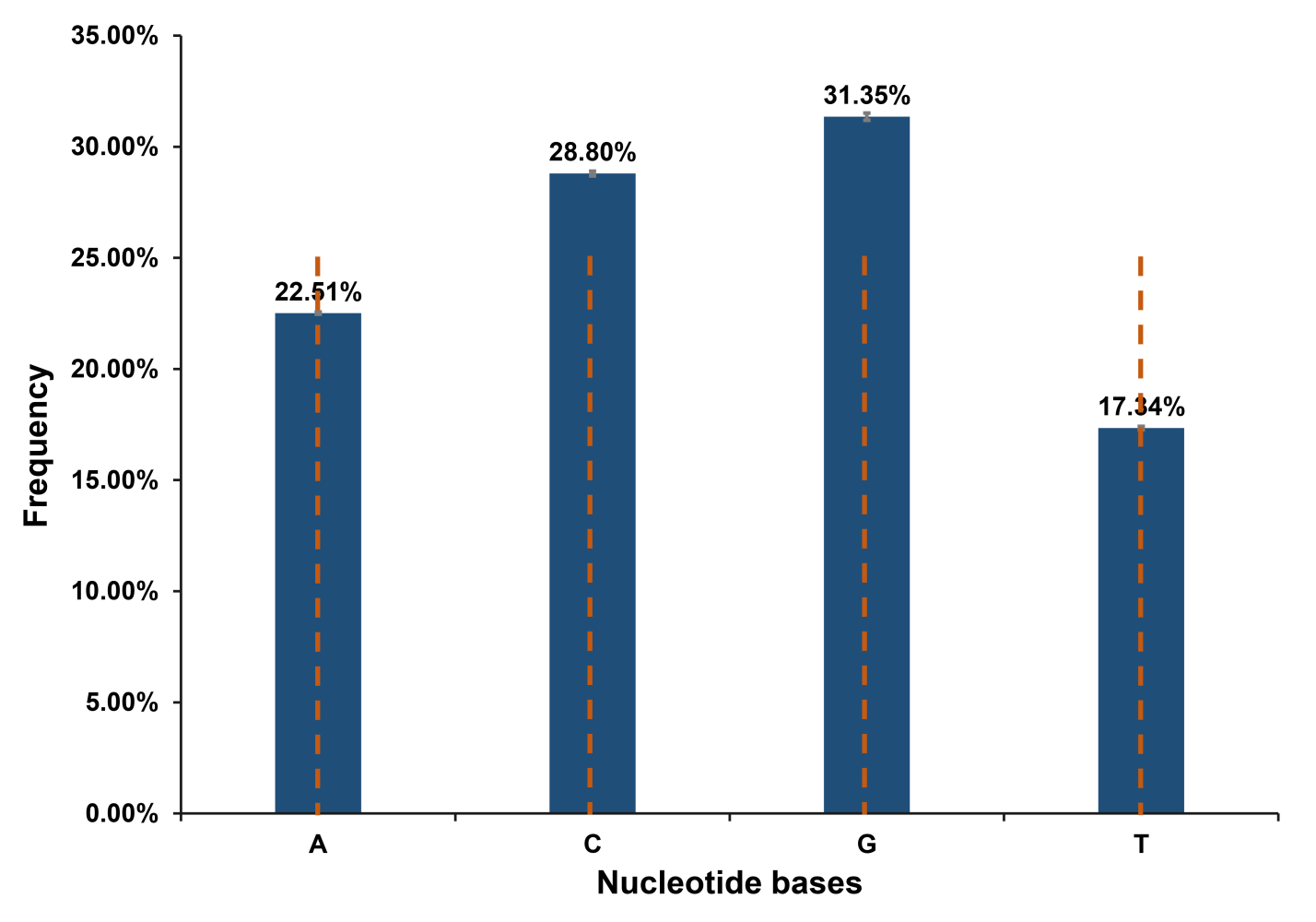


**Fig. S1** Nucleotide composition analysis of the 444-bp *Cyt-b_5_* coding region across 96 *P. infestans* isolates. The histogram displays percentage distributions of adenine (A, 17.34%), thymine (T, 22.51%), cytosine (C, 31.35%), and guanine (G, 28.80%), with error bars representing standard deviations. The dashed line indicates theoretical equal distribution (25% per base). Statistical analysis confirms significant GC bias (60.15% GC content; χ² test, *P* < 0.0001) deviating from random expectation


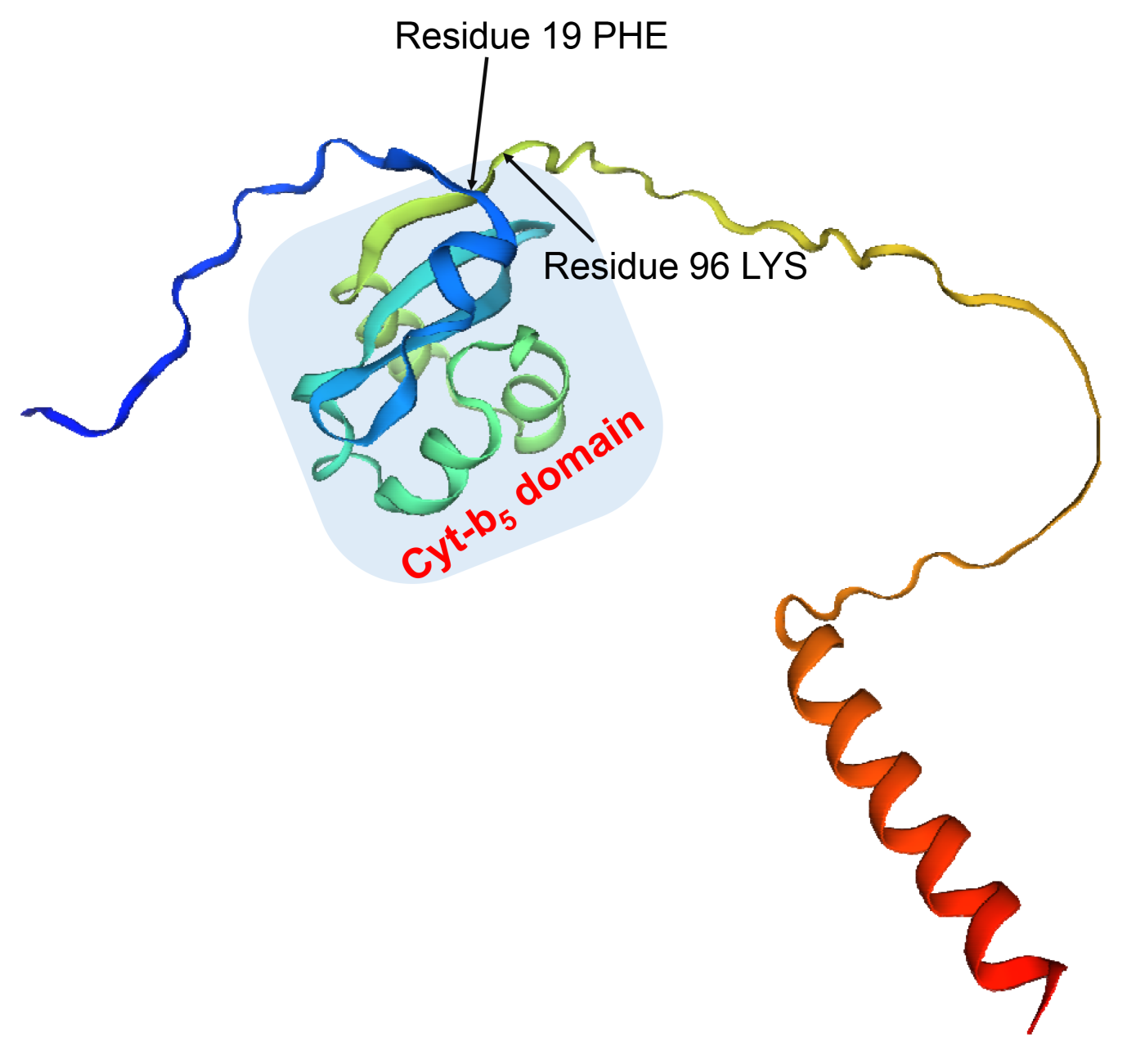


**Fig. S2** Visualization of modeled tertiary structure of Cyt-b_5_ generated by SWISS-MODEL Workspace. The Cyt-b_5_ domain are located in 19 to 96 amino acid residues labeled as a cyan background. 46 PDB ID: 4V7E was having a maximum sequence identity of 19.67%. The used template (AlphaFold DB model of A0A3R7JV96_9STRA) was having a maximum sequence identity of 78.08%


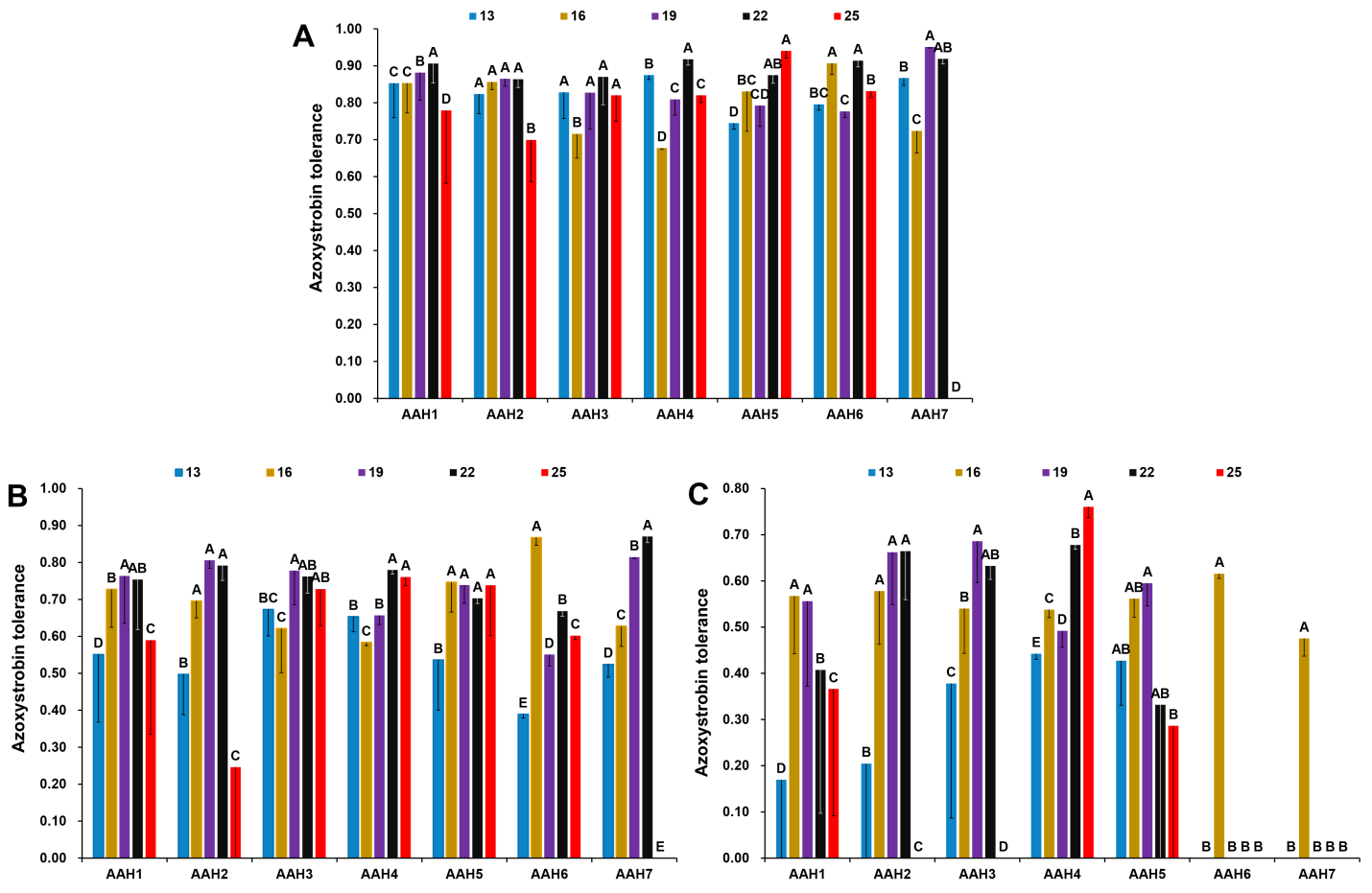


**Fig. S3** Azoxystrobin tolerance of 96 *Phytophthora infestans* isolates sampling from seven geographic locations carrying different Cyt-b_5_ amino acid haplotypes (AAH1-AAH7). A) 0.05 azoxystrobin, B) 0.10 μg/ml azoxystrobin, C) 0.30 μg/ml azoxystrobin. Its error bars were computed across all isolates in each population under the three azoxystrobin concentrations. (0.05, 0.10, and0.30 μg/ml). Duncan’s multiple range test for differences in azoxystrobin tolerance among different Cyt-b_5_ amino acid haplotypes. Values followed by different letters in the same column are significantly different at *P* = 0.05

**
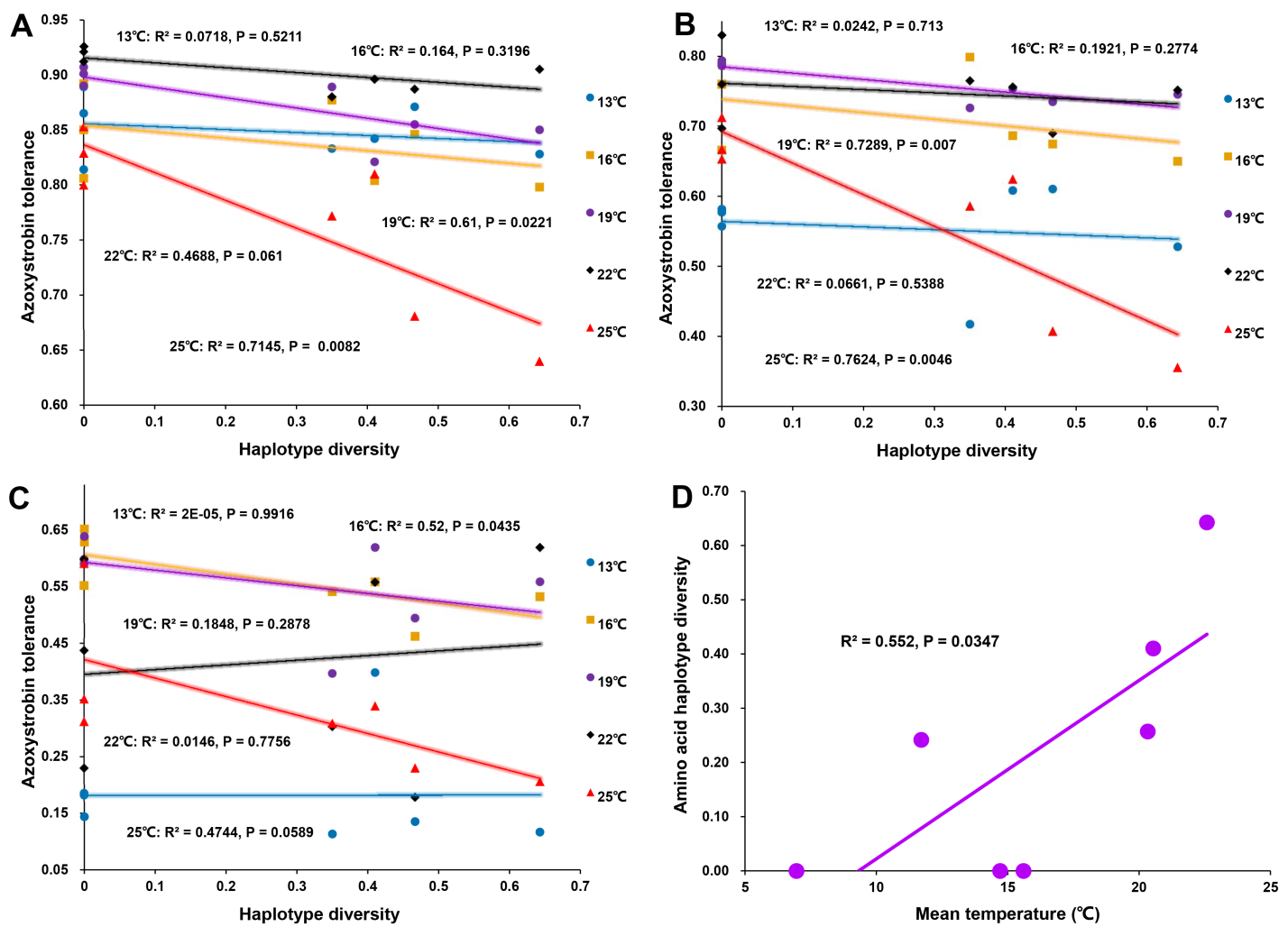
Fig. S4** Bivariate correlations examining relationships between mean annual temperature and Cyt-b_5_ isoform diversity across seven populations. A) 0.05μg/ml azoxystrobin tolerance and *Cyt-b_5_* haplotype diversity, B) 0.10μg/ml azoxystrobin tolerance and *Cyt-b_5_* haplotype diversity, C) 0.30μg/ml azoxystrobin tolerance and *Cyt-b_5_* haplotype diversity, D) mean annual temperature and Cyt-b_5_ amino acid haplotype diversity. Regression lines with Pearson correlation coefficients (r) demonstrate a negative relationship between fungicide tolerance and genetic diversity, while temperature shows positive associations with *Cyt-b_5_* variation
